# Dr. Henry Augustus Gates

**Published:** 1914-08

**Authors:** 


Dr. Henry Augustus Gates, Bellevue, 1877, died at his home in Delhi, May 23, aged 65.

